# Melatonin salvages lead‐induced neuro‐cognitive shutdown, anxiety, and depressive‐like symptoms via oxido‐inflammatory and cholinergic mechanisms

**DOI:** 10.1002/brb3.2227

**Published:** 2021-06-04

**Authors:** Noah A. Omeiza, Halimat A. Abdulrahim, Abdullateef I. Alagbonsi, Precious U. Ezurike, Talha K. Soluoku, Happy Isiabor, Abdulmusawwir A. Alli‐oluwafuyi

**Affiliations:** ^1^ Neuropharmacology and Toxicology Unit Department of Pharmacology and Therapeutics College of Medicine University of Ibadan Ibadan Oyo State Nigeria; ^2^ Department of Medical Biochemistry College of Health Sciences University of Ilorin Ilorin Kwara State Nigeria; ^3^ Department of Clinical Biology (Physiology) School of Medicine and Pharmacy College of Medicine and Health Sciences University of Rwanda Huye Southern Province Republic of Rwanda; ^4^ Department of Neuroscience Faculty of Medicine American University of Beirut Beirut Lebanon; ^5^ Department of Pharmacology and Therapeutics College of Health Sciences University of Ilorin Ilorin Kwara State Nigeria

**Keywords:** anxiety, cholinergic disturbance, cognition, depression, lead‐toxicity, melatonin, neurogenesis, neuro‐inflammation, oxidative stress

## Abstract

**Introduction:**

Lead is the most used nonphysiological neurotoxic heavy metal in the world that has been indicated to interfere with the cognitive and noncognitive processes via numerous mechanisms. The neuroprotective effect of melatonin is well known, but the effect of its interaction with lead in the brain remains inconclusive.

**Objective:**

To assess the therapeutic role of melatonin on cognitive deficit, anxiety and depressive‐like symptoms in matured male Wistar rats exposed to a subchronic lead chloride (PbCl_2_).

**Methods:**

Twenty male Wistar rats were blindly randomized into four groups (*n* = 5/group): group 1 to 4 underwent intragastric administration of physiological saline (10 ml/kg; vehicle), PbCl_2_ (50 mg/kg), melatonin (10 mg/kg) and PbCl_2_ + melatonin respectively for a period of 4 weeks during which neurobehavioral data were extracted, followed by neurochemical and histopathological evaluations.

**Results:**

Exposure to PbCl_2_ reduced cognitive performance by increasing the escape latency and average proximity to the platform zone border, decreasing average path length in the platform zone, cognitive score, and time spent in probing. It raised the thigmotaxis percentage, time spent in rearing, number of pellet‐like feces, and time spent in the dark compartment of a bright/dark box which are predictors of anxiety. It also induced depressive‐like behavior as immobility time was enhanced. PbCl_2_ deranged neurochemicals; malondialdehyde, interlukin‐1β, and tumor necrotic factor‐α were increased while superoxide dismutase and acetylcholinesterase were decreased without remarkable alteration in reduced glutathione and nitric oxide. Administration of PbCl_2_ further disrupted neuronal settings of hippocampal proper and dentate gyrus. In contrast, the supplementation of melatonin reversed all the neurological consequences of PbCl_2_ neurotoxicity by eliciting its properties against oxidative and nonoxidative action of PbCl_2_.

**Conclusion:**

These findings suggest that melatonin down‐regulates neurotoxicant interplays in the brain systems. Therefore, this study suggests the use of melatonin as an adjuvant therapy in neuropathological disorders/dysfunctions.

## Graphical abstract

Melatonin down‐regulates neurotoxicant interplays in the brain systems and its use as adjuvant therapy in neuropathological disorders/dysfunctions is suggested.

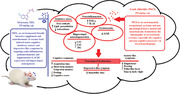

## 1 INTRODUCTION

Lead is a systemic, nonphysiological, and nondegradable toxic heavy metal found in the environment (Ara & Usmani, 2015; Karrari et al., 2012; Sanders et al., 2009 ) and used to produce lead‐acid battery, gasoline, water pipes, paints, solders, ceramic glaze, ammunition, etc. (Ayuso & Foley, 2020; Boldyrev, 2018). It enters the body systems via inhalation of dust, food consumption, and exposure to lead materials (Dignam et al., 2019; Jonasson & Afshari, 2018; Laidlaw et al., 2017 ). Despite extensive studies on the neurological implications of lead and its compounds like lead acetate and lead chloride(PbCl_2_), the nontoxic lead exposure threshold is still unknown.

Lead gets into the systemic circulation through the intestinal mucosa and distributed to various tissues and organs. It crosses the blood–brain barrier and accumulates in the brain tissue to cause neurological dysfunctions (Gąssowska et al., 2016; Li et al., 2016 ). Lead has the ability to disrupt membrane ionic channels and signaling molecules that are crucial in neurotransmission It also inhibits endogenous antioxidants, thereby enhancing the production of reactive oxygen/nitrogen species, leading to oxidative stress (Ayuso & Foley, 2020; Bhat et al., 2015).

Consequently, elevated oxidative stress is implicated in damage to the neuronal polymers (e.g., lipids, proteins, carbohydrates, RNA, and DNA) required in the development and function of the brain (Bhatti et al., 2016).

Furthermore, increasing evidence suggests that nitrosative and oxidative stress are involved in the pathogenesis of neuronal degeneration (Singh et al., 2019). Veritable indicators of neurological disorders (such as Alzheimer's disease, Parkinson's disease, Huntington's disease, and Schizophrenia) include loss of cognitive functions (impairment of attention, learning and memory) (Neylan et al., 2004), anxiety (a condition of worry and nervousness) (Freeman et al., 2012), and depression (a psychiatric syndrome related to a disturbance in mood that is associated with behavioral despair) (Castagné et al., 2011). These indicators are strongly linked to lead toxicity (Ara & Usmani, 2015; Mason et al., 2014 ) and supplements containing antidotal, anti‐oxidant and anti‐inflammatory properties may ameliorate the harmful effects of lead activities in the brain.

Melatonin supplementation has been established to be an environmental‐friendly bioactive molecule with a little biotoxic effect, making it an appropriate supplement for many purposes (Xin et al., 2019; Zhai et al., 2015 ). It is a metabolite of serotonin; a native neuro‐hormone synthesized and released into the brain interstitial fluid, cerebrospinal fluid (CSF), and circulation majorly by the pineal gland (Ruddick et al., 2006) and little from the extra‐pineal tissues (e.g., eye, intestine, skin, platelet, and bone marrow). It peaks at night under normal environmental conditions, transmits the information “darkness” and is suppressed by light (Karrari et al., 2012). It can also be exogenous as a supplement either in the form of synthetic melatonin or phytomelatonin derived from vegetables, seeds, fruits, and nuts (Arnao & Hernández‐Ruiz, 2018a, 2018b). This chemo‐preventive molecule was initially documented to modulate the biological clock and seasonal breeding in living organisms, but further studies have revealed its other pharmacological effects including antioxidant (Olayaki et al., 2018; Reiter et al., 2016; Romero et al., 2014; Srinivasan, 2002), anti‐inflammatory (Chibowska et al., 2020; Permpoonputtana & Govitrapong, 2013; Yu et al., 2017), anxiolytic (Huang et al., 2017; Naranjo‐Rodriguez et al., 2000 ), anti‐depressant (Ramírez‐Rodríguez et al., 2020; Singh & Jadhav, 2014 ), anti‐convulsant (Molina‐Carballo et al., 2007; Muñoz‐Hoyos et al., 1998) and anti‐apoptotic activities (He et al., 2010).

Melatonin is amphipathic in nature, it easily crosses the blood–brain barrier to effect its actions at the higher centers, which can be receptor‐dependent or ‐independent mediated. Melatonin receptors (MT1/MT2) are G‐protein coupled, well distributed in the brain, especially in the hippocampus (a complex of Cornu Ammonis—CA1 to CA4, dentate gyrus, subiculum and the entorhinal cortex) where the cognitive processes are being modulated. Unfortunately, studies have reported lead toxicity affecting some regions of the brain including the cortex and little brain (the cerebellum). A number of studies have correlated low level of melatonin in the plasma and CSF with neurological conditions (Alghamdi, 2018; Kalliolia et al., 2014; Liu et al., 1999; Peres et al., 2006). Therefore, this study was designed to assess the therapeutic role of melatonin on cognitive deficit, anxiety and depressive‐like symptoms in matured male Wistar rats insulted by/with a subchronic lead chloride exposure.

## 2 MATERIALS AND METHODS

### 2.1 Experimental rats

A total of 20 male Wistar rats with an average body weight of 160 ± 20 g (Central Research Animal House, University of Ilorin, Kwara, Nigeria) were used in this study. They were bred and housed in the polystyrene cages under standardized conditions of temperature (25 ± 1°C), relative humidity (60 ± 5%), and photoperiodicity of 12‐hour light/dark starting at 7.00 am. The animals were fed with standard pelletized rodent chow and given potable water ad libitum. They underwent an acclimatization period of 10 days prior to the experimental exposures. All the procedures involving the use of experimental animals were reviewed and approved by the University of Ilorin Animal Care Committee and were in conformity to the criteria outlined in the "Guide for the Care and Use of Laboratory Animals" prepared by the National institutes of Health (NIH) (1986). All efforts were made to establish the principles of the 3Rs of animal research and ensure humane treatment of the experimental animals.

### 2.2 Preparation of drugs and xenobiotic treatments

Melatonin (Sigma‐Aldrich, St. Louis, USA) and lead chloride (Sigma‐Aldrich, St. Louis, USA) used in this study were dissolved in 0.9% normal saline to give individual stock solution from which further dilutions of required concentrations were made and the dosages chosen for the experiment were based on the previous studies on rodent model (Olayaki et al., 2018; Soleimani et al., 2017; Tyagi et al., 2010 ). Lines of evidence show that the efficacy of melatonin is improved with the increased administration and dose of melatonin in human, as doses between 5 and 50 mg daily have been found to improve cognition, sleep and reduce neural injuries (Cardinali et al., 2019). Also, Garza‐Lombó and colleagues (2018) have reported that exposure to lead does not only happen occupationally but also environmentally, some of which are in inorganic form. Hence, inorganic lead such as PbCl_2_ was used in this study to induce neurotoxicity. Having been established to be neurotoxic at low amount, this study was conducted with a higher dose (50 mg/kg) that has been proven to significantly increase toxicity and neural apoptosis (Ahmed et al., 2013; Offor et al., 2017 ). All drugs were freshly prepared and administered via intragastric route in a volume of 1.0 ml per 100 g body weight of rat.

### 2.3 Experimental design

**FIGURE 1:**
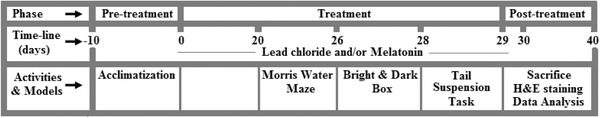
Experimental timeline and design

After 10 days of acclimatization, rats were blindly randomized into four groups (A–D; *n* = 5/group) following an established animal sampling method (Charan & Kantharia, 2013). Groups A to D received normal saline (10 ml/kg), PbCl_2_ (50 mg/kg), melatonin (10 mg/kg), and co‐administration of PbCl_2_ (50 mg/kg) and melatonin (10 mg/kg) respectively. All treatments were given once daily for a period of 1 month between 7:30 am and 9:00 am. Rats were exposed to behavioral tests in Morris water maze (MWM), bright and dark box (BDB), and tail suspension task (TST) 30 minutes after daily treatments (Figure 1). Behavioral performances were done between 10:00 am and 4:00 pm, recorded by a video camera (Webcam) mounted directly above the mentioned models’ apparatus. Trajectory and navigation parameters in the maze were tracked and analyzed using ANY‐Maze video tracking system software from Stoelting Co. (Version 6.3; Wood Dale, IL). Twelve hours prior to the sacrifice, rats were fasted, and on the 30th day (between 8:00 and 10:00 am), rats were euthanized by quick cervical dislocation. Brain tissues were excised and processed for neurochemical assay and histopathological evaluations (Figure 1).

### 2.4 Behavioral evaluations

#### 2.4.1 Morris water maze task

Morris water maze is a popular paradigm test used to assess neurophysiological and pathological processes that alter spatial and nonspatial learning and memory including the anxiety‐like behavior in experimental rodents. The apparatus composed of a circular pool of diameter 1.80 m and height 0.60 m, filled with opaque water, 0.40 m deep at a controlled temperature of 25 ± 1°C. The pool was then divided into north, east, south, and west zones with a circular platform of diameter 0.10 m, submerged 0.02 m beneath the water surface and placed at the center of the northeast zone, 0.15 m from the perimeter of the pool. Prior to the spatial training trials, rats were allowed to acclimatize to the water maze pool without platform. After 24 hours, we commenced the task by placing individual rat in the southwest zone facing the wall of the pool to navigate and locate the hidden platform that serves as an escape route from a pool of water within a maximum swimming time of 60 seconds. The rats that found the hidden platform were allowed to remain and explore the terrain for 30 seconds, then dried with absorbable towels and placed back into its home plastic cage, while those that failed to locate the platform were guided by the experimenter. The spatial training trials were performed twice per day with an intertrial interval of 30 minutes for four consecutive days as earlier described (Salomon et al., 2011). The hallmark for this task is acquisition; therefore, it is also termed acquisition trial.

Twenty‐four hours after the last trial, four probe tests were conducted with 30‐minute intertrial interval. During the 1st trial, rats were allowed to swim freely without the platform for 60 seconds in order to explore the platform zone habitually. Parameters like average proximity to the platform zone border, latency time to explore, and path length in the platform zone were recorded to measure spatial retention, learning, and memory. On the subsequent trials, the platform was replaced to its original training position in the pool and the latency to escape in the second trial is a measure of long‐term memory while the average latency to escape in the 3rd and 4th trials measures the short‐term memory. The last three trials performed on this day were used to determine cognitive performance by scoring each trial across the groups using the scale: 1 = thigmotaxis; 1 = passivity; 2 = random; 3 = circling; 3 = chaining; 3 = accidental circling; 4 = focused; 5 = corrected; 6 = direct, as described in the previous studies (Higaki et al., 2018; Illouz et al., 2016). In evaluating the anxiety‐like response of the rats in MWM paradigm, thigmotaxis (which is the percentage of time spent in navigating the wall of the maze within 0.15 m distance) and the time spent in rearing during the 2nd day of spatial training session were recorded.

#### 2.4.2 Bright and dark box task

Twenty‐four hours after the probe test, rats were exposed to BDB exploration. This model is used to predict the anxiety‐like behavior and cognitive deficits in rodents. The apparatus composed of an opened doorway (0.10 × 0.10 m) in the partition wall that connected two equally divided chambers of a box (0.46 × 0.27 × 0.30 m). The dark chamber was painted black with a black plastic roof, while the other chamber had transparent walls without roof. In the course of habituation, an individual rat was placed in the center of bright chamber facing the opened doorway and allowed to explore the chambers freely for 10 minutes, twice per day with 30‐minute intertrial interval for 2 days. The box was thoroughly cleaned with methylated alcohol and dried before placing the next rat on it; this curtailed the biasness due to odor cues left by the previous rat. After 24 hours, the opening was blocked with a black plastic door and the time spent by the rat in probing the door and the number of its pellet‐like feces dropped during the test were recorded to evaluate the memory consolidation process and autonomic function or anxiety‐like behavior of the rats exposed to lead and/or melatonin.

#### 2.4.3 Tail suspension task

On the 29th day of the experiment, rats underwent tail suspension task. This model is used for the assessment of depressive‐like behavior in rodents. In order to establish the test, individual rat's tail was wrapped with an adhesive tape in a constant position approximately 1 cm from the tip of the tail. A hook was suspended through the adhesive tape on a thin rod supported at the ends by two retort stands, placed 50 cm above the floor. Rats were observed continuously for 6 minutes and the immobility time, a measure of depression was well defined as the total time spent by the rats hanging passively.

### 2.5 Brain tissue preparation for neurochemical assays and histopathological evaluations

On the 30th day and 12‐hour postprandial exposure, rats were sacrificed by quick cervical dislocation and perfused transcranial with 30 ml of physiological saline followed by 50 ml phosphate buffer saline. The whole brain tissue was quickly excised, rinsed in 0.25 M sucrose ice at 4°C and dissected into two hemispheres. The right hemisphere was homogenized with mechanized homogenizer (LANKAI FSH 2A, CHINA) in 30% chilled sucrose in 1:4 ratio w/v; centrifuged at 4000 rpm for 30 min at 4°C to residue and the resulting supernatants on the top were aspirated into plain bottles and stored at −20°C prior to neurochemical assays, while the left hemisphere was fixed in 10% formol saline for 72 h. Thereafter, the tissues were processed and stained with hematoxylin and eosin (H and E) for histopathological examination.

### 2.6 Neurochemical assays

Determination of neurochemical concentrations or activities was performed on the supernatant of brain homogenates using spectrophotometric technique. The activities of brain redox enzymes superoxide dismutase (SOD) and reduced glutathione (GSH) were determined by the method of Kakkar et al. (1984) and Moron et al. (1979) respectively. The brain malondialdehyde (MDA) was performed using a thiobarbituric acid reactive substance for measuring the peroxidation of fatty acids (Ohkawa et al., 1979) and the nitric oxide (NO) level was estimated based on the conversion of nitrate to nitrite via nitrate reductase and formation of purple azo compound as previously described by Guevara et al. (1998). The concentrations of brain cytokines; interleukin (IL‐1β) and tumor necrosis factor‐alpha (TNF‐α) were evaluated in accordance with the manufacturers’ description in the assay kit packs (Elabscience Biotechnology Co., Ltd., Wuhan, PR China). Furthermore, the activity of acetylcholinesterase (AChE) was estimated by the modified method of Ellman et al. (1961).

### 2.7 Hematoxyllin and eosin staining and quantitative morphometric study

The fixed brain tissues were cleaned in xylene and processed to obtain paraffin wax embedded in paraffin, refrigerated and sectioned into 5‐μm sections of the hippocampal blocks using arotary microtome (MK 1110), and stained with H and E for general cytoarchitecture following the standard protocol for histology (Imam et al., 2016). Thereafter, stained slides were mounted, and histological evaluation was conducted to observe and analyze the degree of neuromorphological distortions in the hippocampal regions of CA1, CA3, and dentate gyrus of rats treated with lead and/or melatonin using a light microscope (Nikon, Tokyo, Japan) at 400× magnification with an in‐built camera attached to a monitor to produce photomicrographs. Prior to the microscopic view of the slides, the slides were coded and an experimenter was used to blind the rat's condition in order to avoid biasness during all phases of image collection and analysis. For quantitative estimation, Image J analyzer software (NIH Image, Maryland, USA) was used to count the total number of neuronal cells of hippocampal proper (CA1 and CA3), and suprapyramidal blades of dentate gyrus. Quantitative data were collated for statistical analysis and values were presented in terms of mean ± SEM for cells/area in mm^2^ (*n* = 3/group).

### 2.8 Statistical analyses

GraphPad Prism 8.0 (GraphPad Software Incorporated, La Jolla, CA) was used to analyze behavioral, neurochemical and morphometric data. Comparisons among the groups were done by one‐way analysis of variance (ANOVA) while that between the treatment group and day was carried out using a repeated measures two‐way ANOVA followed by post hoc Tukey's multiple tests, respectively. Values were expressed as mean ± SEM of the variables measured. Statistical significance was considered at *p* < .05.

## 3 RESULTS

### 3.1 Melatonin supplementation prevented lead‐induced neurocognitive deficits

**FIGURE 2:**
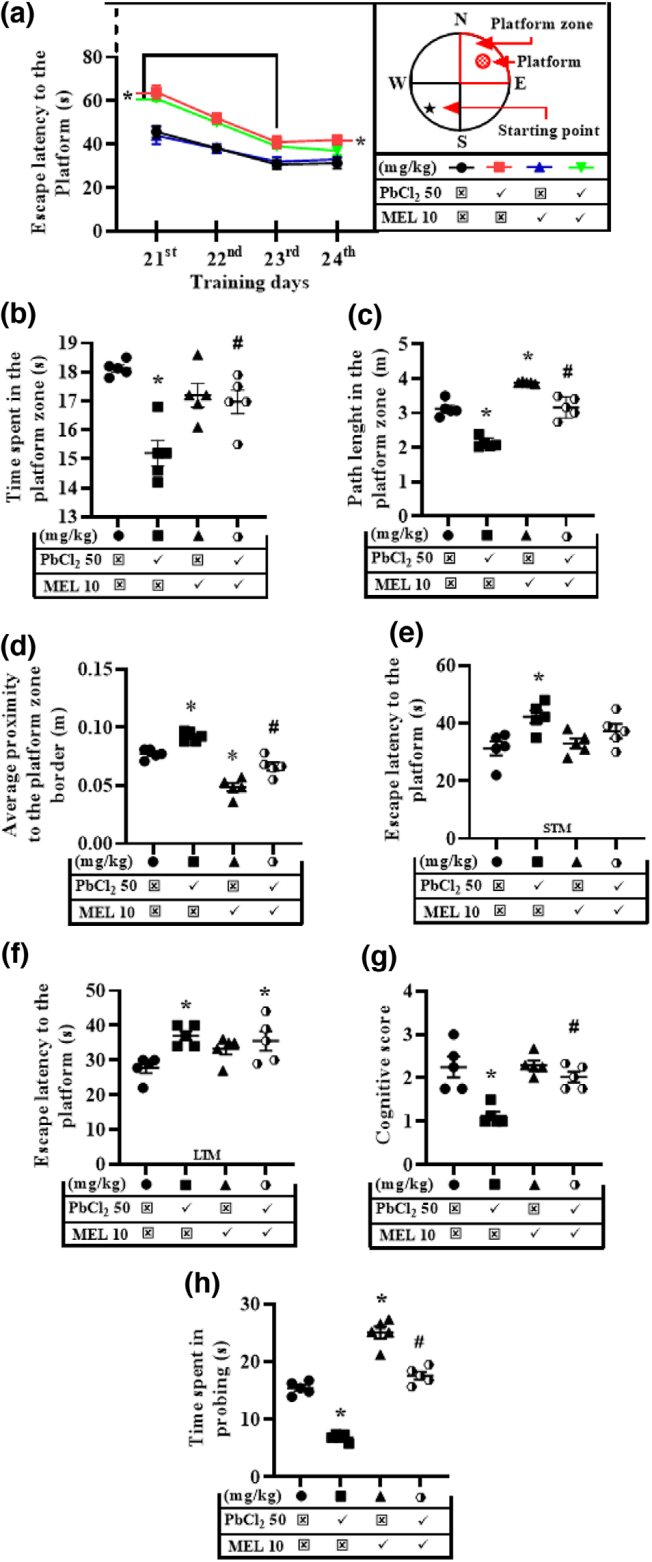
Representative results for the Morris water maze and ‘bright and dark box’ tasks illustrating the multitasking role of melatonin on lead‐induced cognitive deficit. The following parameters were accessed: In the MWM—Escape latency (a) showing the acquisition trial (days 1–4); time spent in the platform (b); path length in the platform zone (c); average proximity to the platform zone border (d); escape latency for the short (e) and long‐term memory (f); and cognitive score (g) during the probe trial on the 5^th^ day. In the BDB—time spent in probing (h) was evaluated on the test day. PbCl_2_, lead chloride; MEL, melatonin; *n* = 5/group, ^*^significantly different from the control group at *p* ≤ .05, ^#^significantly different from PbCl_2_ (50 mg/kg) group at *p* ≤ .05

Spatial learning and memory defined the neurocognitive function of the brain and they were evaluated in MWM and BDB tasks. In the MWM task during the acquisition trial (training day), escape latency (the time taken to locate the hidden platform) decreased over the first three days and stabilized on the fourth day of learning across all the groups, showing that all the animals learned to find the hidden platform (Figure 2(a)). Two‐way ANOVA (group × day) for repeated measures indicated that each of the treatments had a significant effect on the escape latency. The group (F3, 64 = 30.88, *p* < .0001) and the day (F3, 64 = 45.49, *p* < .0001) effects on escape latency were both significant respectively but the interaction between the group and day effects was considered insignificant (F9, 64 = 1.372, *p* = .2196). Furthermore, Tukey post‐hoc analysis showed that the escape latency was increased on the 21^st^, 22^nd^, and 23^rd^ day of acquisition trial in lead (64.50, 52.88, 41.25 s) and lead + melatonin‐treated (61.5, 50.63, 39.75) groups when compared to control (45.75, 38.25, 30.75 s) respectively. However, on the 24^th^ day of acquisition trial, melatonin prevented this effect of lead as the escape latency was increased by lead treatment (42.25 s) alone but unchanged by lead + melatonin (37.25 s) treatment relative to control (31.25 s). Besides, the escape latencies of melatonin‐treated group (44.00, 38.03, 32.05, 33.00 s) remained unchanged when compared with the control (45.75, 38.25, 30.75, and 31.25s) throughout the observation period (Figure 2(a)).

During the probe trial on the 25th day, reference or retention memory was accessed in all groups without the platform in the pool. One‐way ANOVA and Tukey post‐hoc analysis revealed that the time spent (18.13 ± 0.12 s vs. 15.20 ± 0.44 s) and the path length (3.12 ± 0.10 m vs. 2.13 ± 0.07 m) in the platform zone were decreased and average proximity to the platform zone (0.077 ± 0.001 m vs. 0.092 ± 0.002 m) on other hand was increased by lead treatment when compared with control. When administered alone, melatonin did not affect the time spent (17.20 ± 0.4 s) but increased the path length (3.88 ± 0.01 m) and decreased the average proximity to (0.048 ± 0.004 m) the platform zone. Interestingly, melatonin abolished the effect of lead by maintaining the time spent (16.97 ± 0.41 s), path length (3.16 ± 0.14 m) and average proximity to (0.067 ± 0.004 m) the platform zone at values comparable to the control (Figure 2(b)—(d)).

Short‐term memory (STM) was evaluated with the platform present in its zone and the escape latency was increased in lead‐treated (42.25 ± 21.2 s) but unchanged in melatonin‐treated (33.00 ± 1.70 s) rats when compared to the control (31.25 ± 2.48 s). However, the lead‐induced increase in escape latency time was abolished by melatonin in rats that received both treatments (37.25 ± 2.457 s) (Figure 2(e)).

For long‐term memory (LTM) assessment with the platform, escape latency was also increased in lead‐treated (37.00 ± 1.34 s) but unchanged in melatonin‐treated (33.25 ± 1.62 s) rats when compared to the control (27.75 ± 1.50 s). Quite the reverse, the lead‐induced increase in escape latency time was unchanged by melatonin in rats that received both treatments (35.50 ± 2.80 s) (Figure 2(f)). Taken together, lead caused reduction in the cognitive score of rats (1.13 ± 0.10), which was abolished by melatonin in rats that received their combination (2.02 ± 0.12), maintaining the score around that of the control (2.25 ± 0.24) (Figure 2(g)).

In the BDB task, the time spent in probing was decreased by lead (6.85 ± 0.29 s) but increased by melatonin (25.06 ± 1.05 s), while melatonin abolished the lead‐induced reduction in the probing time in rats that received their combination (17.60 ± 0.66 s) by maintaining it at a value comparable to that of the control (15.44 ± 0.51 s) (Figure 2(h)).

### 3.2 Melatonin supplementation abrogates lead‐induced anxiety‐like symptoms in rats

**FIGURE 3:**
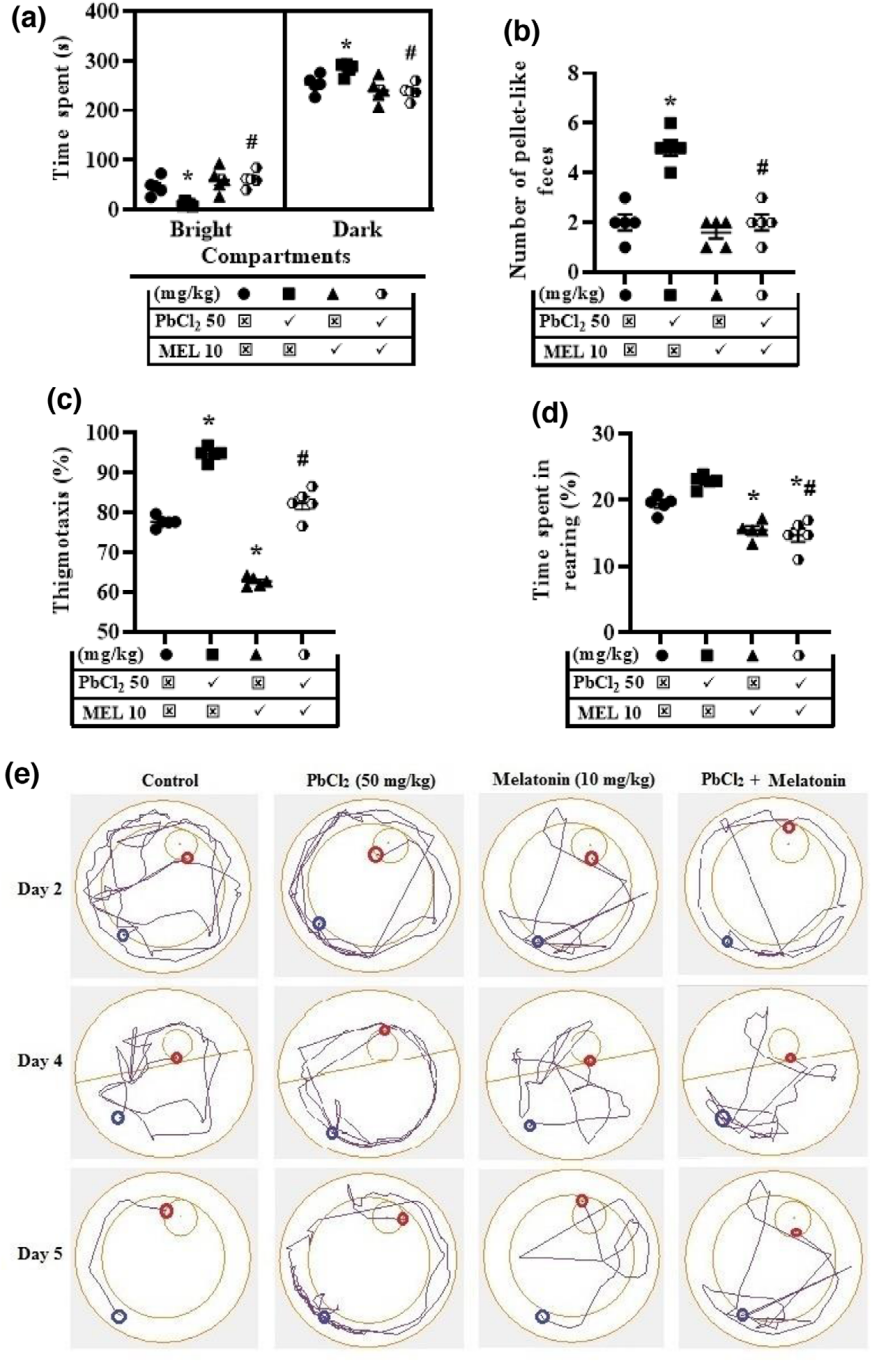
Representative results for the bright and dark box and Morris water maze tasks showing the reversal effects of melatonin on lead‐induced anxiety‐like behavior. The following parameters were accessed: In BDB—time spent in bright and dark compartments (a) and number of pellet‐like feces (b). In MWM—thigmotaxis (c) and time spent in rearing (d) during the acquisition trial (day 2) were all accessed. Micrographs depicting the swimming trajectories of the rats during the evaluation of anxiety‐like responses and cognitive processes (acquisition, consolidation or retention) when platform was in the pool on the 2nd, 4th, and 5th day for the task (e). Values are expressed as mean ± SEM. Data were analyzed using analysis of variance and Tukey post hoc test for multiple comparisons. *n* = 5/group. ^*^significantly different from the control at *p* ≤ .05, ^#^significantly different from PbCl_2_ (50 mg/kg) group at *p* ≤ .05. PbCl_2_; lead chloride, MEL; melatonin, blue and red circles indicate the starting and end points in MWM respectively

The effect of melatonin on lead‐induced anxiety‐like symptoms in rats was evaluated using the BDB and MWM tasks. In the BDB task, lead‐treated rats showed anxiety‐like symptom as there was an increase (284.0 ± 5.36 s) in the time spent in dark compartment of bright/dark box when compared to control (253.5 ± 7.99 s). While the time spent by melatonin‐treated rats was insignificantly reduced (240.3 ± 10.78 s), melatonin further abolished the lead‐induced anxiety‐like symptom in rats that received their combination (238.3 ± 7.15 s) by maintaining the time spent in the dark compartment around the control value (Figure 3(a)). Similarly, the number of pellet‐like feces dropped in the arena was higher in lead‐treated rats (5.00 ± 0.32) than the control (2.00 ± 0.32), and the increase was abolished by melatonin in rats that received their combination (2.00 ± 0.32s) (Figure 3(b)).

In the MWM task, the thigmotaxis was elevated by lead (94.77 ± 0.75%) but decreased by melatonin (62.69 ± 0.53%) treatments when compared to control (77.68 ± 0.61%), but the increase by lead was abolished by melatonin (82.35 ± 0.63%) (Figure 3(c)). Furthermore, the time spent in rearing was increased in lead‐treated rats (22.79 ± 0.42%) relative to control (19.40 ± 0.58%) but decreased in melatonin (15.44 ± 0.60%) and lead + melatonin‐treated (14.70 ± 1.02%) groups (Figure 3(d)).

The trajectory swimming activities of the rats in all treated groups on the 2^nd^, 4^th^, and 5^th^ day revealed the expediency of MWM as a model in assessing anxiety‐like symptom and cognitive deficits using the thigmotaxis and cognitive score respectively. The level of thigmotaxis, swimming distance, and time declined over the course of the time in all groups except that of lead‐treated group (Figure 3(e)).

### 3.3 Melatonin supplementation ameliorates lead‐induced depressive‐like symptoms in rats

**FIGURE 4:**
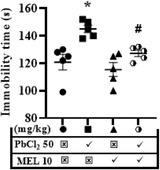
Representative results for the tail suspension task illustrating the effect of melatonin on lead‐induced depressive‐like symptom—immobility time. Values are expressed as mean ± SEM. Data were analysed using analysis of variance and Tukey post hoc test for multiple comparisons. *n* = 5/group. ^*^Significantly different from the control at *p* ≤ .05, ^#^significantly different from PbCl_2_ (50 mg/kg) group at *p* ≤ .05. PbCl_2_; lead chloride, MEL; melatonin

The effect of melatonin on lead‐induced depressive‐like symptoms in rats was assessed using TST. Generally, lead treatment (145.0 ± 2.72 s) enhanced the immobility time when compared to control (120.8 ± 5.62 s). However, this increase was abolished by melatonin in rats that received their combination (127.2 ± 2.23 s) (Figure 4(a)).

### 3.4 Melatonin supplementation reverses lead‐induced neurochemical derangement in rats

Oxidative stress status (MDA, SOD, GSH, and NO), pro‐inflammatory cytokines (IL‐1β and TN F‐α), and cholinergic pathway (AChE) biomarkers were assessed to establish the effects of melatonin on lead‐induced neurochemical derangement in the brain.

**FIGURE 5:**
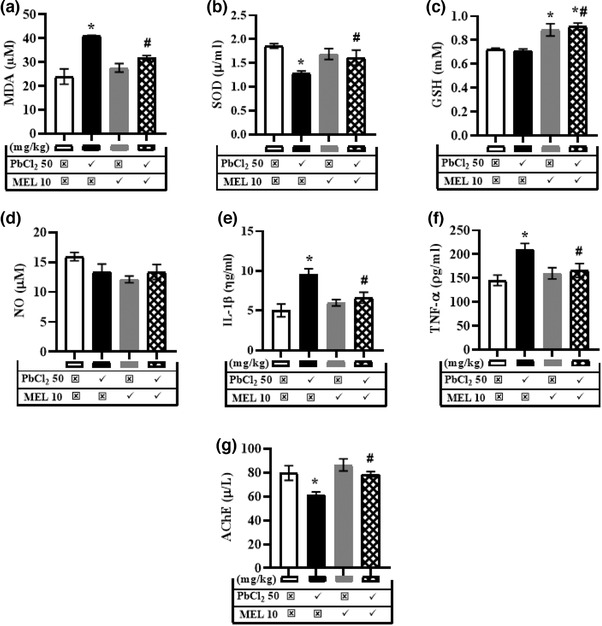
Representative results showing the reversal effects of melatonin on lead induced neurochemical derangement in rats. The following parameters were accessed: MDA (a), SOD (b), GSH (c), NO (d), IL‐1β (e), TNF‐α (f) and AChE (g). Values are expressed as mean ± SEM. Data were analyzed using analysis of variance and Tukey post hoc test for multiple comparisons. *n* = 5/group. ^*^Significantly different from the control at *p* ≤ .05, ^#^significantly different from PbCl_2_ (50 mg/kg) group at *p* ≤ .05. PbCl_2_; lead chloride, MEL; melatonin, MDA; malondialdehyde, SOD; superoxide dismutase, GSH; reduced glutathione, NO; nitric oxide, TNF‐α; tumor necrotic factor‐α, IL‐1β; intelukin‐1β, AChE; acetylcholinesterase

The MDA level, a product of lipid peroxidation in the cell membrane, was elevated by lead (41.23 ± 0.07 μM) but unchanged by melatonin (27.61 ± 1.77 μM) when compared to control (23.92 ± 2.04 μM). However, melatonin abolished the lead‐induced increase in MDA in rats that received their combination (32.37 ± 2.99 μM) (Figure 5(a)).

The SOD activity was decreased by lead (1.28 ± 0.05 μm/ml), which was abolished by melatonin in rats that received their combination (1.61 ± 0.16 μm/ml) by maintaining it around the control value (1.86 ± 0.05 μm/ml) (Figure 5(b)).

The GSH activity was increased in rats that received melatonin alone (0.89 ± 0.05 mM) and its combination with lead (0.92 ± 0.03 mM), but not in those that received lead only (0.71 ± 0.01 mM) when compared to control (0.73 ± 0.01 mM). (Figure 5(c)).

The NO level was not affected by lead and/or melatonin (Figure 5(d)).

TNF‐α concentration was elevated in the lead‐treated (210.5 ± 12.01 ρg/ml) group when compared to control (145.4 ± 10.95 ρg/ml) but reduced in melatonin‐(160.0 ± 11.82 ρg/ml) and lead+melatonin‐treated (166.0 ± 14.64 ρg/ml) groups (Figure 5(e).

Furthermore, IL‐1β level was elevated in lead‐treated (9.55 ± 0.73 ηg/ml) group when compared to control (5.03 ± 0.81 ηg/ml) group, but lowered in melatonin (6.00 ± 0.41 ηg/ml) and lead+melatonin‐treated (6.63 ± 0.69 ηg/ml) groups (Figure 5(f)).

Lastly, the AChE activity was reduced by lead (61.41 ± 2.22 μm/L), which was abolished by melatonin in rats that received their combination (78.58 ± 2.156 μm/L) when compared to control (79.47 ± 6.12 μm/L) (Figure 5(g)).

### 3.5 Melatonin supplementation reverses lead‐induced hippocampal degeneration in rats

**FIGURE 6:**
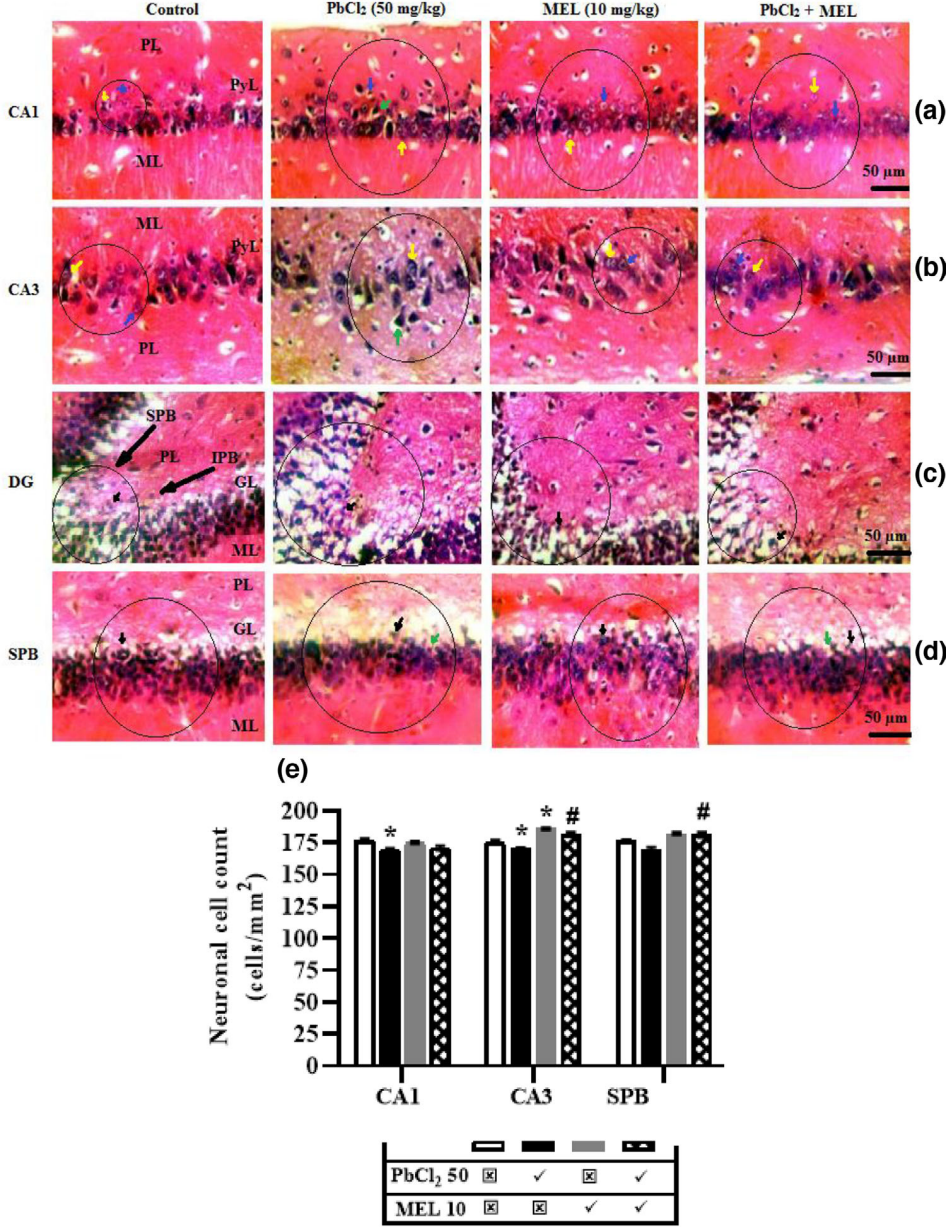
Representative results depicting hematoxylin and eosin (H&E) staining and quantitative neuronal cell count in the hippocampal regions of CA1, CA3, dentate gyrus and suprapyramidal blade of dentate gyrus. Melatonin reversed lead‐induced histopathological distortion in CA1 (a), CA3 (b), DG (c) and SPB (d). For quantitative analysis, melatonin ameliorates neuronal cell count decline in the hippocampal subfields induced by lead (e). The slides (*n* = 3) were viewed by light microscopy; magnification: × 400; scale bar = 50 μm. Values are expressed as mean ± SEM. Data were analyzed using analysis of variance and Tukey post hoc test for multiple comparisons. *n* = 3/group. ^*^Significantly different from the control at *p* ≤ .05, ^#^significantly different from PbCl_2_ (50 mg/kg) group at *p* ≤ .05. PbCl_2_; lead chloride, MEL; melatonin, CA1; CA3; DG; SPB; blue line; pyramidal cell, yellow line; vesicular cell, green line; neuronal degeneration (vacuolization and atrophy), black line; granular cell

The hippocampal CA1 and CA3 subfields in lead‐treated group had pyramidal cells that were not well organized in a single layer between molecular and polymorphic layers, and showed degradation of the nuclear material resulting to vacuolization of the cells while the vesicular cells’ nuclei showed multiple fragmentation of the nuclear materials, thereby appearing fenestrated when compared to control group that appeared normal, well‐organized layer with active nuclear material in the pyramidal cells and pale staining round‐shaped nuclei of the vesicular cells that formed the cellular arrangement of the hippocampal subfields (CA1 and CA3). Melatonin and lead + melatonin treated groups showed normal active pale‐staining nuclei of vesicular cells and pyramidal cells that were deeply purple/blue stained in comparison to control group. However, no traces of vacuolization of the cells were observed, indicating the normal functioning of the nuclei which maintain the integrity of the cells. Also, the pyramidal cells layer is most densely packed in CA1, and less densely packed in CA3 across all the treated groups (Figure 6(a) and (b)). Figure 6(c) revealed the V‐shape or the converging point of suprapyramidal and infrapyramidal blades of DG. In lead‐treated group, granule cell (or principal cell) layer at the converging point was not densely packed with granular cells when compared to control group. However, these cells were amplified in melatonin‐treated group and the effect of lead appeared to minimize in lead + melatonin‐treated group in comparison to the lead‐treated group. Furthermore, there was a severe atrophy of clusters of granule cells located on the suprapyramidal part of the DG in lead‐treated group when compared to control group. This effect of lead was reduced by melatonin as a moderate atrophy of clusters of cells were observed in lead + melatonin‐treated group when compared to control (Figure 6(d)).

Figure 6(e) showed the effect of lead and/or melatonin on the neuronal cell count of hippocampal CA1, CA3, and DG (SPB). The neuronal cell count of CA1 in lead‐treated (169.7 ± 1.01 cells/mm^2^) group decreased when compared to control (177.2 ± 1.13 cells/mm^2^) group, which was abolished by melatonin in rats that received their combination (171.0 ± 1.69 cells/mm^2^). In CA3, the neuronal cell count was likewise lowered by lead (170.5 ± 0.73 cells/mm^2^) but increased by melatonin (186.7 ± 1.93 cells/mm^2^) when compared to control (175.6 ± 1.61 cells/mm^2^). This effect of lead was reversed (*p <* .05) by melatonin in lead + melatonin‐treated (182.1 ± 1.19 cells/mm^2^) group when compared to lead‐treated group. Furthermore, lead exposure (169.3 ± 1.19 cells/mm^2^) insignificantly decreased the neuronal cell count of suprapyramidal blade of dentate gyrus when compared to control (176.2 ± 1.15 cells/mm^2^) but increased by melatonin in rats that received their combination (181.1 ± 2.10 cells/mm^2^).

## 4 DISCUSSION

Lead toxicity is associated with brain oxidative stress, neuro‐inflammation, and excitotoxicity. Currently, the interaction among these factors is considered as the hallmark of the neurologic structural and functional derangement in the brain after lead exposure. The hippocampus, being an important neurobiological area for cognitive processes, is believed to be central to the deleterious effects of lead. Melatonin possesses antioxidant, free radical scavenging, anti‐apoptotic and anti‐inflammatory properties. The administration of varying doses of lead in experimental animals has been used widely to investigate the sequelae of lead exposure and a battery of behavioral paradigm tests including the MWM, BDB, open arm maze, active and passive avoidance tests have been effectively used to study the effects of lead on neurocognitive processes and behavior (McNamara & Skelton, 1993; Nehru & Sidhu, 2002). We investigated the therapeutic potential of melatonin to salvage lead‐induced neuro‐cognitive deficits, anxiety and depressive‐like symptoms, and hippocampal degeneration in matured male Wistar rats. We further explored the anti‐oxidative role of melatonin vis‐à‐vis its neuroprotection against lead‐induced behavioral derangement. This study reports that lead exposure impairs learning, induces anxiety and elicits depressive‐like symptoms in lead‐exposed male Wistar rats. In the MWM test, which is used to evaluate the acquisition of spatial learning and memory, lead‐exposed rats exhibited a significant increase in escape latency, a decrease both in time spent and path length in the platform and an increase in average proximity to the platform zone—all indicators of cognitive impairments which may lead to difficulties in reference memory as a result of hippocampal functional deficits (Gonenc et al., 2005; Soleimani et al., 2016). Melatonin reversed the lead‐induced deficits in cognitive performance as evidenced by the increase in time spent and path length in the platform zone while decreasing escape latency and average proximity to the platform zone in the melatonin and lead‐melatonin treated groups. These findings are consistent with previous findings that melatonin treatment was able to improve cognitive performance in a model of hippocampus‐dependent spatial learning and memory impairment (Gonenc et al., 2005; Lee et al., 2016; Tuzcu & Baydas, 2006).

The effects of lead were also assessed on the short‐ and long‐term memory of the animals. The study shows lead‐induced impairment in the working memory and retentive (explicit) memory of the experimental rats as there exist an increase in escape latency in both memory models. The lead‐induced increase in escape latency was abolished by melatonin. The cognitive score was also assessed as an overall prediction of cognitive functioning, as changes in memory and other mental functions may occur leading to impairments which tend to lower the cognitive performance (score) (Dalrymple‐Alford et al., 2010). The observed reduction in cognitive score in the lead‐treated group as well as the melatonin‐induced improvement of same as seen in melatonin‐lead treated group suggest that melatonin supplementation plays a role in lead‐induced neuronal impairment.

Time spent in probing (explorative behavior), a measure to further assess the neurocognitive functions of spatial learning and memory in the brain, was evaluated using BDB task. Our study suggests that lead decreased the time spent in probing, which might have resulted from memory deficit associated with cognitive alterations. However, melatonin abolished the lead‐induced reduction in probing time, thereby increasing the explorative behavior. Hence, the ability of melatonin supplementation to reverse the lead‐induced behavioral impairment may be attributed to its amphipathic nature as it easily crosses the blood–brain barrier to modulate learning and memory centers on the hippocampal functions (Rawashdeh & Maronde, 2012).

An increased number of fecal boli is associated with anxiety of the animals (Flint et al., 1995). In this study, lead exposure induced anxiety‐like behaviors as evidenced in the increase in the number of fecal boli (emotionality), time spent in rearing response (motor activity) and also increase in thigmotaxis (habituation‐ rapid movement away from the central habitual area to the wall of the field). However, melatonin improves the lead‐induced anxiety‐like behavioral symptoms in the experimental rat model. This finding suggests that lead, which can easily be absorbed through the intestinal mucosa of the gastro‐intestinal (GI) tracts before gaining entrance into systemic circulation where further distribution takes place (Marchetti, 2003), may have accumulated and caused damage to the endothelial cells. The damage could have resulted to abnormal intestinal lesions and barrier function, thereby increasing motility which is evident in the anxiety‐like symptoms and other physiological effects in the hippocampus affecting parasympathetic movement. These anxiety‐like symptoms, as revealed, were all improved by melatonin as evidenced in the decrease in the number of fecal boli, time spent in rearing and duration of thigmotaxis. Melatonin, though majorly produced by the pineal gland, is also presumably produced in high amount in the entero‐endocrine cells of GI mucosa (extra‐pineal sources), as the main enzymes of melatonin synthesis, arylalkylamine N‐acetyltransferase and hydroxyindole‐*O*‐methyltransferase have been detected in GI mucosa (Bubenik, 2008; Stefulj et al., 2001). This wide synthesis underlines its diverse physiological roles which may be affected by lead exposure and thus a melatonin supplementation will augment and restore its physiological normalcy through process of cell proliferation and differentiation (Hill et al., 2009). Hence, the observed effects of melatonin treatment are its ability to restore and maintain the integrity and functionality of cellular membranes as it can interact with lipid bilayers as evidenced in decrease in GIT motility and increase in mucosal blood flow (Bubenik, 2008; Lu et al., 2009) and its antioxidant activity in GI system (De Talamoni et al., 2011).

Furthermore, this study reports the amelioration of lead‐induced depressive‐like symptoms by melatonin. The TST was used to monitor the depressive‐like behavior induced by lead treatment and it is based on the assumption that immobility reflects a measure of behavior despair (Crowley et al., 2005). In TST, animals were placed in an inescapable but moderately stressful situation. Lack of escape‐related behavior was considered immobility, thus a measure of depressive‐like symptom. In our study, it was observed that exposure to lead resulted in an increase in immobility time. This is suggested as a sign of depression as the animals’ inability to handle stress increases. Depression is often viewed as a lack of ability to handle/cope with stress, hitherto, associated with disability, decreased quality of life and increased health‐related cost (Sullivan et al., 2000). Melatonin supplementation was able to ameliorate the depressive‐like symptom by a decrease in immobility time. The improved stress condition will potentially increase the struggling and escape behavior of the animal and its ability to handle stress (Cryan et al., 2005). This suggests that melatonin may be used as a mild antidepressant or as an adjuvant therapy to improve depression‐like conditions.

An imbalance between the production of reactive oxygen species (ROS) and the ability of the antioxidant systems to readily detoxify these reactive intermediates results in oxidative stress. Elevated oxidative stress has been implicated in a wide variety of neurodegenerative disorders (Gelderman et al., 2007; Hwang, 2013). Lead damages cellular components via elevation of oxidative stress as it directly interrupts the activity of enzymes, deactivating or depleting antioxidants sulfhydryl pools and free‐radical induced damage through direct formation of ROS (Ercal et al., 2001; Patrick, 2006). This study revealed that melatonin completely attenuates the lead‐induced increase in the levels of MDA (a lipid peroxidation product) and restored GSH level and SOD activity. This finding is consistent with the report of El‐Sokkary et al. (2003). The neuro‐protective effect of melatonin in lead‐treated group is related to its reported direct radical‐scavenging actions, indirect antioxidant effects and stimulatory effect on anti‐oxidative enzyme activities in arresting oxidative stress‐induced neuronal damage (Rehman et al., 2019; Reiter et al., 2009). SOD, a class of biological enzyme is developed as an important antioxidant agent in living cells that promotes the disproportionation of superoxide into oxygen and hydrogen peroxide, which is then rapidly decomposed by enzyme catalase to oxygen and water (Löffler & Petrides, 1988). This suggest that a decreased SOD activity will lead to a decrease in the rate of disproportionation of superoxide, which resulted into a decrease in the formation of hydrogen peroxide. Melatonin also demonstrates a direct detoxification of free radicals and an indirect anti‐oxidative effect that potentiates lead‐induced oxidative stress in the cerebellum (Bazrgar et al., 2015). Moreover, melatonin is able to be converted by reactive oxygen species into a more stable cyclic 3‐hydroxymelatonin, which is even more potent than melatonin itself. This metabolite, which prevents mitochondrial cytochrome C injury and reduces apoptosis, is speculated to be the means by which melatonin mediates its antioxidant function (Tan et al., 2014).

Furthermore, lead‐mediated toxicity has been shown to elevate cytokines, which implicate neuro‐inflammation (Bhat et al., 2019). The observed lead‐induced increase in the concentration of TNF‐α and IL‐1β may be associated with the induction of neuroinflammation‐induced memory impairment (Fernandez et al., 1998; Kim et al., 2014). Oxidative stress triggers inflammatory response by activating the microglia and astrocyte as effector cells to release several pro‐inflammatory cytokines in cascade of events which increase the release of TNF‐α leading to inflammation and thus promoting neurodegeneration (Smith et al., 2012; Wang et al., 2006). Neuronal death increases following inflammation and TNF‐α exacerbates the degeneration process. In this study, we observed that melatonin treatment reduces the over‐production of IL‐1β and TNF‐α, and prevents neuroinflammation and memory impairment in the lead‐induced experimental rats. It can be deduced that melatonin reversed lead‐induced neuroinflammation by downregulation of cytokines. This further validates the immunomodulatory and anti‐inflammatory properties of melatonin (Bonnefont‐Rousselot et al., 2011; Guerrero & Reiter, 2002; Wu et al., 2014).

Earlier studies have shown that lead poisoning affects multiple target systems in the brain as it may cause impairments in the cholinergic system associated with behavioral dysfunctions and deficits in learning and memory, since these neurotransmitters are believed to regulate motor coordination and emotions (Reddy et al., 2003, 2007). The levels of acetylcholine are continuously regulated by the hydrolytic enzyme acetyl cholinesterase (AChE), which rapidly degrades acetylcholine both in the periphery and in the brain. Therefore, we measured AChE activity in brain as a marker of cholinergic activity. In the present study, lead exposure significantly decreased the activity of membrane‐bound enzyme AChE, significantly inhibiting its activity. Lead has affinity for the sulfhydryl groups in enzymes and proteins and its binding can alter their function (Bagchi et al., 1997; Chetty et al., 1990). Lead has also been reported to stimulate the production of free radicals leading to elevated oxidative stress by inactivating antioxidant enzymes. All these may be reasons for the decreased AChE activity in lead‐exposed rats. However, the co‐administration of melatonin restored the AChE to a normal level in the lead‐exposed group. Melatonin has been reported to scavenge free radicals thereby offsetting oxidative stress (Karbownik et al., 2001), reduce inflammatory response (Lee et al., 2007), reduce blood brain barrier permeability (Chen et al., 2006), improved neuronal survival and enhance neurogenesis (Kilic et al., 2008). All these properties may attribute to lead‐induced inhibition of AChE activity. Acetylcholine level increases in response to a decrease in the activity of AChE. Studies on some brain regions have indicated the spontaneous release of neurotransmitters by micro molar concentration of lead (Nehru & Sidhu, 2002; Shao & Suszkiw, 1991). Therefore, we can speculate that lead exposure induces neurobehavioral disturbances by altering cholinergic neurotransmission as melatonin supplementation restored AChE activity to its normal level as observed from the study.

Another significant finding was the reversal of lead‐induced hippocampal degeneration in experimental rats by melatonin supplementation. It was observed that lead treatment resulted in an unorganized, degraded and multiple fragmented nuclear materials (cellular components) in the pyramidal and vesicular cells of the hippocampus CA1 and CA3 neuronal subfields. The effect was also seen in the decrease in the neuronal density of granular cells as well as the severe atrophy that insulted the clustered granular cells on the suprapyramidal segment (SPG) in the dentate gyrus (DG) of the hippocampal formation. This result correlates with reported studies showing maximal level of lead accumulation occurring significantly in the hippocampal region of the brain causing structural damage to the brain cell components through elevated oxidative stress and inhibition of DNA repairs (Beyersmann & Hartwig, 2008). In some cases, it inhibits the absorption of important trace minerals necessary for neuronal survival and its involvement in direct formation of ROS while interrupting enzyme activity (Boldyrev, 2018; Patrick, 2006). All these evidences compromise the neuronal architecture that triggers hippocampal damages which gradually lead to loss of neuronal functions, decrease in synaptic activities and spatial learning and memory impairments (cognitive deficits) as a result to neurodegeneration (Farkas et al., 2007; White et al., 2007).

However, melatonin treatment significantly reversed the lead‐induced effects as no vacuolated cell was traced, neuronal density was increased, and moderate atrophy was observed in the examined hippocampal block and thus further maintaining the integrity of the cell for normal hippocampal functioning of the brain. In addition, lead‐induced an appreciable increase in neuronal cell death (decreased neuronal cell count) of hippocampal CA1, CA2 and SPG of DG while melatonin supplementation was able to reverse the lead‐induced neuronal cell death in the hippocampal regional block of the melatonin‐lead group. This validates the previous reports that melatonin treatment attenuated neuropathological alterations in the hippocampus (El‐Sokkary et al., 2003; Ozacmak et al., 2009). The increase in neuronal cell count in both the hippocampal CA1, CA3, and the SPG of DG mediated by melatonin treatment on lead‐induced neuronal cell death therefore shows the ability of melatonin to protect against lead‐induced spatial learning and memory impairment (cognitive deficits) and neurodegeneration, which may be associated with the neuro‐protective effects of melatonin in these hippocampal regional areas under study.

**FIGURE 7:**
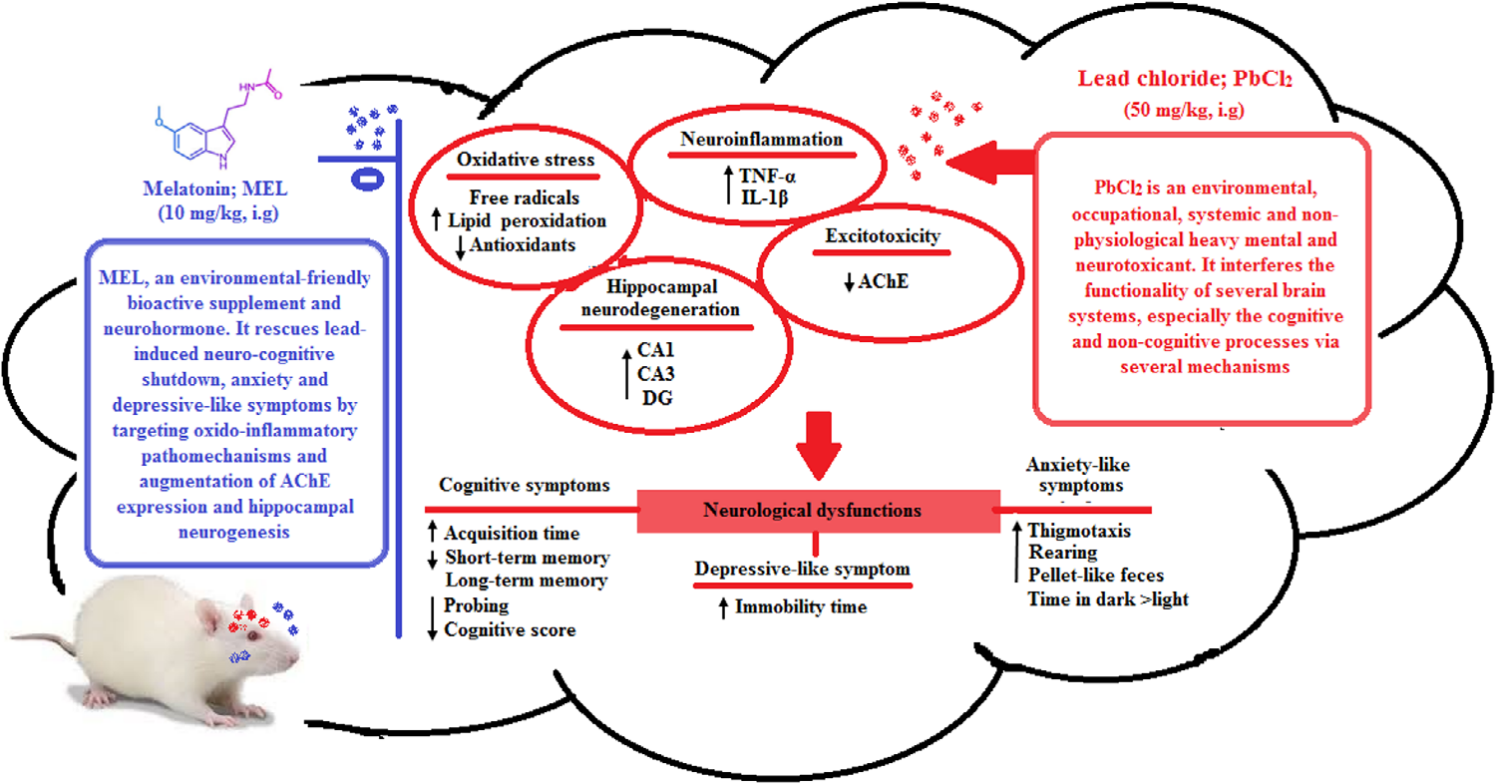
Graphical abstract illustrating the probable pathways through which melatonin supplementation targeted lead‐induced neuro‐cognitive shutdown, anxiety and depressive‐like symptoms

In summary, the data in the present study support the observation that lead results in impaired memory acquisition, anxiety and depressive‐like symptoms which are associated with oxidative stress, neuro‐inflammation, cholinergic disturbance and structural derangements particularly in the hippocampus. However, melatonin prevents/ameliorates the lead‐induced behavioral, neurochemical and morphological changes in lead‐exposed rats (Figure 7).

One of the limitations associated with this study is the inability to assay the neurochemical components (such as the brain‐derived neurotrophic factor alpha, nuclear factor kappa B and caspases) to further validate the neuroprotective actions of melatonin. We were also unable to establish the melatonin receptor subtype that plays a significant role in attenuating lead‐induced neurotoxicity. Also, there was no recording of the lead content of the animal blood and cerebellum, which would have enabled us to understand the extent of lead accumulation and relate it to the observed parameters in the experimental groups. These will be our focus in the future studies so as to better understand the protective role of melatonin against lead‐induced neurotoxicity.

## AUTHORSHIP CREDITS

N.A.O: concept/design, acquisition of data, data analysis and interpretation, drafting of the manuscript; H.A.A: supervision, concept/design, acquisition of data; A.I.A: data analysis and interpretation, drafting/critical revision of the manuscript; P.U.E: acquisition of data, drafting of the manuscript; T.K.S: drafting of the manuscript; H.I: acquisition of data; A.A.A: critical revision of the manuscript. All authors revised and reviewed the manuscript.

## CONFLICT OF INTERESTS

Authors declared that no conflict of interest.

### PEER REVIEW

The peer review history for this article is available at https://publons.com/publon/10.1002/brb3.2227.
